# Dr. John Kendrick Wynne

**Published:** 1911-09-16

**Authors:** 


### OBITUARY.

The death is announced of Dr. John Kendrick Wynne, M.D., late of Eccleshall, Staffordshire.

